# Nonlinear Detection for a High Rate Extended Binary Phase Shift Keying System

**DOI:** 10.3390/s130404327

**Published:** 2013-03-28

**Authors:** Xian-Qing Chen, Le-Nan Wu

**Affiliations:** School of Information Science and Engineering, University of Southeast, Nanjing 210096, China; E-Mail: wuln@seu.edu.cn

**Keywords:** support vector machine, low density parity check codes, extended binary phase shift keying, posterior probability, intermediate frequency demodulator

## Abstract

The algorithm and the results of a nonlinear detector using a machine learning technique called support vector machine (SVM) on an efficient modulation system with high data rate and low energy consumption is presented in this paper. Simulation results showed that the performance achieved by the SVM detector is comparable to that of a conventional threshold decision (TD) detector. The two detectors detect the received signals together with the special impacting filter (SIF) that can improve the energy utilization efficiency. However, unlike the TD detector, the SVM detector concentrates not only on reducing the BER of the detector, but also on providing accurate posterior probability estimates (PPEs), which can be used as soft-inputs of the LDPC decoder. The complexity of this detector is considered in this paper by using four features and simplifying the decision function. In addition, a bandwidth efficient transmission is analyzed with both SVM and TD detector. The SVM detector is more robust to sampling rate than TD detector. We find that the SVM is suitable for extended binary phase shift keying (EBPSK) signal detection and can provide accurate posterior probability for LDPC decoding.

## 1. Introduction

Increasing demand for wireless communication in various areas of human life has brought about an exponential increase in the number of wireless services. There will be a continuous increase in the demand for wireless spectrum in the foreseeable future with the introduction of internet multimedia applications such as online video, multimedia networks, and distributed gaming. This exponential increase has resulted in spectrum scarcity as the electromagnetic spectrum has become too crowded to incorporate all the upcoming wireless services. There is more of an increase in demand for spectrum than for development in technology, which aims at increasing the spectrum efficiency [1,2]. Meanwhile, due to growing demands for wireless multimedia services and the rollout of advanced radio transmission technologies wireless networks are consuming increasing amounts of energy and contribute a growing fraction to the CO_2_ emissions of the information and communications technology industry. There is a need on environmental grounds to reduce the energy requirements of wireless communications [3], thus the energy consumption and energy efficiency issue is another research focus of the wireless communications research community.

Recently, these problems have attracted a lot of attention of researchers and many new ideas have been proposed to mitigate the problem of spectrum scarcity and energy efficiency [4–8]. In order to satisfy the higher and higher demand for communication systems, a new technique called efficient modulation that can achieve high data rates and high spectrum efficiency is receiving the attention of many researchers [9,10]. In this research, another modulation technique referred to as extended binary phase shift keying (EBPSK) which has better bandwidth efficiency, and higher data rates was proposed by Wu *et al.* [11]. The difference between the waveforms of EBPSK modulation corresponding to “0” and “1” is very tiny, if we use classical theory (such as by using the matched filter or correlation detection) to detect the signals, it has even higher demand on input SNR. Fortunately, a special impacting filter (SIF) which can produce high impact at the phase jumping point and great improvement in SNR, was applied at the receiver [12]. Therefore, a simple amplitude detector could perform the detection of EBPSK signals [13]. However, due to the characteristics of EBPSK modulation, if we increase the bit rate by using the short bit duration, the SIF output signals will interfere with the neighboring symbols which is the phenomenon of intersymbol interference (ISI) [14]. Furthermore, most transmission systems have band limitations imposed by either the natural band-width of the transmission medium or by regulatory conditions. If a narrowband band-pass filter (NBPF) is added at the transmitting end of the communications system to achieve a bandwidth-efficient transmission, the ISI is also hard to avoid, so it is difficult to detect the received signals by threshold decision (TD), because the signal amplitude of SIF output is no longer high [15]. Meanwhile, nonlinear detectors are specifically designed to get the optimum performance of blind multi-user detectors [16], and nonlinear channel equalization [17] and provide accurate posterior probability estimates for LDPC decoding [18]. All results have shown that the nonlinear detection technique performs like an optimum receiver. One of the goals of this paper is the analysis of a nonlinear detector based on support vector machine (SVM), together with the SIF. Although, the SVM approaches are less principled than Gaussian processes classification (GPC), it is impractical when the number of training samples of the GPC is not very low [19], so in this paper, we focus on the SVM technique for EBPSK signal detection and posterior probability estimates (PPEs).

Some preliminary works on detecting the signal of SIF output have been presented in [13,20,21]. All of these systems were concentrated on reducing the bit error rate, instead of increasing the data rate, reducing energy consumption and providing accurate posterior probability estimates that can be exploited by a soft-input channel decoder to achieve capacity.

The contribution of this paper is to cover high bit rate, low bit error rate (BER) and low energy consumption by applying the SVM technique. A numerical example is used in giving a brief demonstration of the SVM detector and the design parameters have been considered and investigated for the purpose of optimization and simplification. Other related issues such as the kernel selection, features extraction and reducing complexity of the detector have also been analyzed. In addition, we give the analysis of state-of-the-art nonlinear detector together with the channel decoder.

The remainder of this paper is organized as follows: Section 2 is devoted to introducing the efficient modulation. We present the receiver scheme in Section 3 and briefly describe the SVM classification and PPEs for LDPC decoding. In Section 4, we include illustrative experiments to compare the performance of the SVM detector. We conclude in Section 5 with some final comments.

## 2. Efficient Modulation

The increasing demand for frequency resources is becoming a tough problem when allocation and reallocation of frequency bandwidth are periodically repeated. Higher level modulations are used in solving the problem. However, these solutions are all at the expense of increased energy. Furthermore, the order grows in powers of two, while the constellation is becoming dense and difficult to divide. As a result, only binary modulation or binary keying make sense to easily and fairly measure the bandwidth efficiency.

Efficiency modulation was first proposed by Walker, who holds several patents on the technique. After his cooperation with Photron Science Company, these patents were registered as ultra spectral modulation (USM), which has pretty high bandwidth efficiency. In some other literatures, from a signal bandwidth rather than power bandwidth point of view, such a system can be referred to as a carrier-synchronized ultra-wide band system (CS-UWB) [22], which can produce a high narrow carrier spectrum when the practical duty-cycle is low.

**Figure 1.**
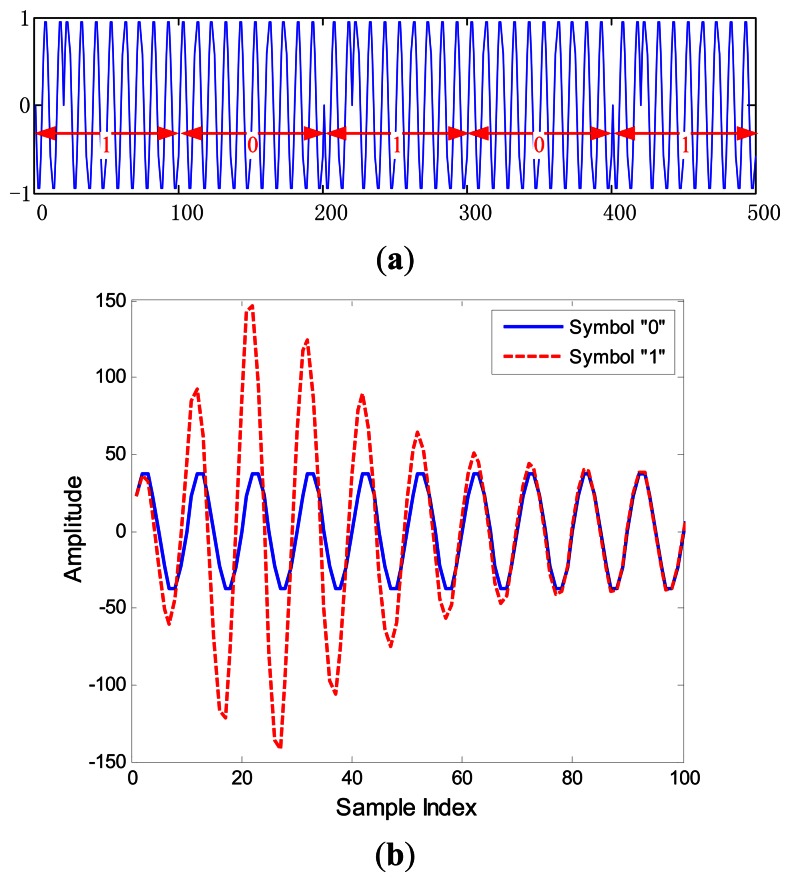
EBPSK modulation with *N* = 10, *θ* = *π*, *K* = 2, *A* = *B* = 1 in (**a**) and SIF output with symbol “0” and “1” in (**b**).

From a unified expression perspective, all of these techniques are actually a special EBPSK system that is defined as follows:

(1) $$\begin{array}{l} \begin{array}{cc} g_{0}(t)=A\sin2\pi f_{c}t, & 0\leq t<T \end{array}\\ g_{1}(t)=\{\begin{array}{cc} B\sin(2\pi f_{c}t+\theta ), & 0\leq t<\tau ,0\leq \theta \leq \pi \\ A\sin(2\pi f_{c}t), & \tau \leq t<T \end{array} \end{array}$$

where *g*_0_ and *g*_1_ are the modulation waveforms corresponding to bit “0” and bit “1”, respectively; *T* = *N*/*f_c_* is the bit duration, *τ* = *K*/*f_c_* is the phase modulation duration, *f_c_* is the carrier frequency, and *θ* is the modulating angle. If we set *τ* = *T* and *θ* = *π*, then Equation (1) becomes the classical binary phase shift keying (BPSK) modulation. Moreover, T controls the bit rate, (*i.e.*, the bit rate with N = 5 is 4 times than with N = 20), so if we want to increase the bit rate, we can use a short bit duration N. As an example, Figure 1(a) below is the waveform of EBPSK modulation and Figure 1(b) is the waveform of SIF output.

According to the FCC's bandwidth definition, there should be a total 99% signal power hold in the band. Such a power reservation criterion is practically equivalent to the −20 dB attenuation bandwidth, indicating that spectral attenuation from the peak power to the cutoff frequency point is no less than 20 dB. The −40 dB attenuation bandwidth of EBPSK modulation is only several Hertz [11]. In order to full understand the characteristics of EBPSK modulation, its power spectra should be analyzed. According to reference [20], the power spectrum density (PSD) of EBPSK can be written as follows:

(2) $$\begin{array}{l} s(f)=\frac{1}{8T\pi^{2}{\left(f_{c}^{2}-f^{2}\right)}^{2}}\left[\left(A^{2}f_{c}^{2}+B^{2}f_{c}^{2}\cos^{2}\theta -2ABf_{c}^{2}\cos\theta +B^{2}f^{2}\sin^{2}\theta \right)\cdot (1-\cos2\pi f\tau )\right]\\ +\frac{1}{16T^{2}}\delta \left(\;f-\frac{N}{T}\right)\left[\tau^{2}(A^{2}+B^{2})+{4T}^{2}A^{2}-4T\tau A^{2}+4ABT\tau \cos\theta -2AB\tau^{2}\cos\theta \right]\\ +\frac{1}{8T^{2}\pi^{2}}\sum_{\begin{array}{l} m=-\infty \\ m\neq N \end{array}}^{\infty }\delta \;\left(f-\frac{m}{T}\right)\frac{1}{{\left[f_{c}^{2}-{\left(\frac{m}{T}\right)}^{2}\right]}^{2}}\cdot \left(1-\cos2\pi \frac{m}{T}\tau \right)\;.\\ \left\{\left[A^{2}f_{c}^{2}+B^{2}f_{c}^{2}\cos^{2}\theta -2ABf_{c}^{2}\cos\theta +B^{2}{\left(\frac{m}{T}\right)}^{2}\sin^{2}\theta \right]\right\} \end{array}$$

where *m* is integer and the other parameters are defined as in (1). The PSD of EBPSK is made up of a continuous part and a discrete part. The width of the mainlobe and sidelobe in the continuous part is controlled by 2*πfτ*. The interval of the discrete part, exhibited as linear spectra, is influenced by the factor 1/T. In this paper we focus on the high bit rate of the system, and so the short bit duration T (or N) should be used. Moreover, the linear spectra containing no information can be removed with some optimization and the phase shift remains in the waveform with amplitude changed but no infection [23].

## 3. Detection in Receiver

### 3.1. Threshold Decision

The difference between the waveforms of “0” and “1” is very small, so a traditional IIR or FIR filter with a narrow bandwidth can erase the minute difference information and leave only a sine wave, such that we cannot perform detection in the receiver [12]. Therefore, a well designed SIF which can separate the tiny difference of the received waveforms must be used in solving this problem. The transfer function of the proposed SIF can be written as follows:

(3) $$H(\omega )=\frac{1+b_{1}e^{-j\omega }+b_{2}e^{-2j\omega }}{1+\sum_{i=1}^{2n}a_{i}\cdot e^{-ij\omega }}$$

where n is the pair number of the conjugate poles.

**Figure 2.**
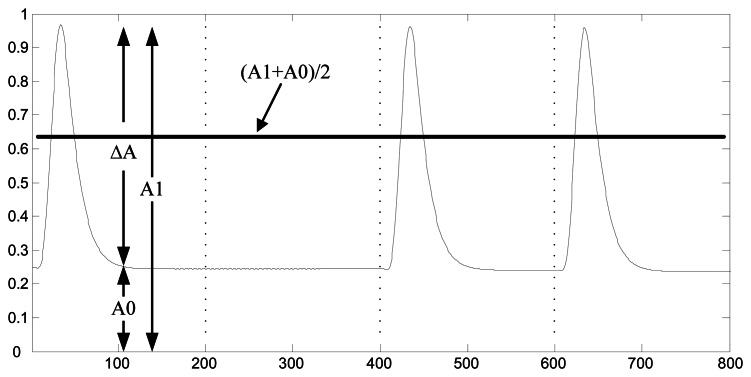
The threshold decision with σ^2^ = 0.

Therefore a simple amplitude detector can be used in separating the symbols “0” and “1” because of the existence of high impulse in coded 1 s. From reference [13] we can get the optimal threshold if the symbols only interfered with AWGN. The threshold can be obtained as follows:

(4) $$u_{T}=\frac{1}{2}\left(A_{1}+A_{0}+\frac{2\sigma^{2}}{A_{0}-A_{1}}\text{ln}\sqrt{\frac{A_{0}}{A_{1}}}\right)$$

where the σ^2^ is the noise variance, A0 and A1 is the maximum amplitude of the filter output corresponding to code “0” and “1”, respectively, as is shown in Figure 2.

According to reference [11], the value of *A*_0_ and *A*_1_ can be obtained through the following equations:

(5) $$A_{0}=A\cdot \left|H(\omega_{c})\right|=\sqrt{2E_{b}/T}\cdot \left|H(\omega_{c})\right|$$

and:

(6) $$A_{1}=A_{0}+\Delta A$$

where the value of Δ*A* can be obtained in Reference [13].

**Figure 3.**
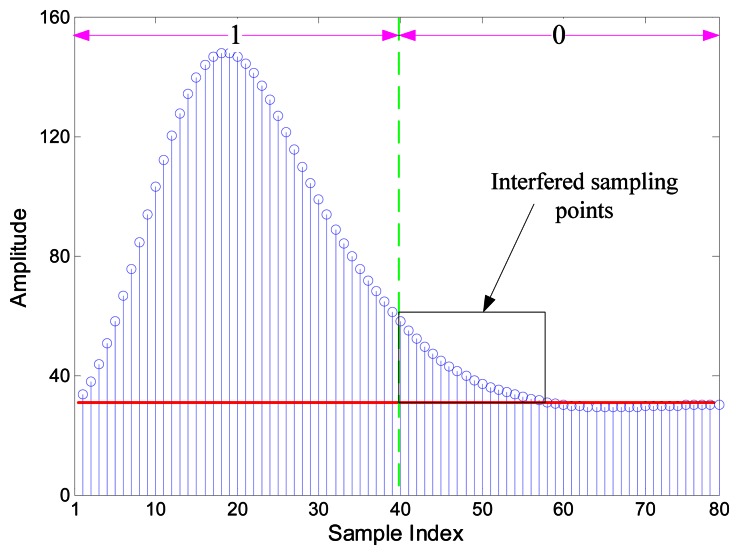
The envelop of symbol “1” and its followed symbol “0” with bit duration N = 4.

Though the SIF transforms phase modulation into amplitude changes, several signal cycles that followed the change part are distorted. Obviously, if we use short bit duration, the subsequent symbol will be interfered with as is shown in Figure 3. The phase shift is influenced and so the impacted amplitude of the following symbol would be not as high as the one with long bit duration.

**Figure 4.**
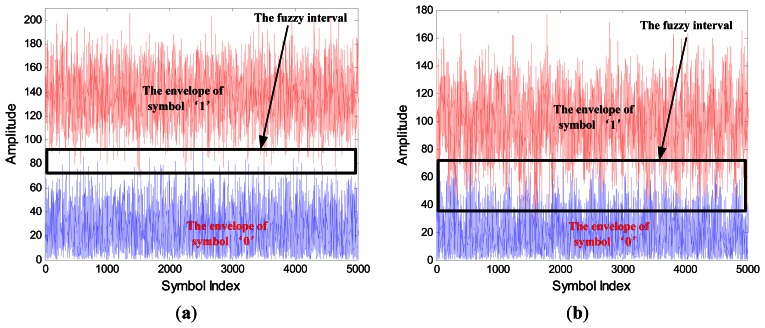
The signals envelope of SIF output with SNR = −2 dB, N = 20 in (**a**) N = 5 in (**b**).

Figure 4 shows the signals envelope of SIF output with N = 20 in (a) and N = 5 in (b). If we use a short bit duration N, then ISI occurs, and the fuzzy interval between symbol “0” and “1” is large.

Also, the signal amplitude of symbol “1” with short bit duration is lower than that with long bit duration, but for symbol “0” both of them are almost identical. According to the relationship between *A*_0_ and *A*_1_ in (6), we know that if the Δ*A* is low by using short bit duration, it becomes difficult to detect the amplitude by TD.

### 3.2. SVM Detector

In this section, we suggest a nonlinear detection algorithm from an appealing pattern classification point of view. We detect the received signals of SIF output by using the SVM technique. The main advantage of using such a technique is that it can make full use of the characteristics of the received waveforms.

#### 3.2.1. SVM Classification

For the binary classification problem, during the training stage, the goal of SVM is to seek a separation plane which maximizes the margin between the two classes of 1 and 0. Each input training sequence ***x****_i_* ∈ **R***^n^*, *i* = 1, 2, …, *L* is associated with a binary message *y_i_* ∈ {1,0} to indicate the desired output. After training is completed, the decision function is constructed via:

(7) $$f(x)=\sum_{i=1}^{L}\alpha_{i}y_{i}K(x,x_{i})+b$$

where L is the number of training sequence, *α_i_* is a Lagrangian constant which contributes to the slope of the separation plane, *K*(**x**, **x_i_**) = Ψ(**x_i_**)^T^ Ψ(**x**) is a kernel function, where Ψ(**x**) maps the training data vector **x**_i_ into the high-dimensional feature space, and b is a bias term of the decision hyperplane.

Define a coefficient vector **w**, such that:

(8) $$w=\sum_{i=1}^{L}\alpha_{i}y_{i}\psi (x_{i})$$

then the training is completed by solving the following optimization problem:

(9) $$\underset{\text{w}\in \text{H},b\in \text{R},\xi \in {\text{R}}^{L}}{\min}\frac{1}{2}{\left\|\text{w}\right\|}^{2}+C\sum_{i=1}^{L}\xi_{i},\text{s.t}\text{.},y_{i}((\text{w}\cdot {\text{x}}_{i})+b)\geq 1-\xi_{i},\xi_{i}\geq 0,i=1,2,...,L.$$

where *C* is the trade-off parameter between the training error and the margin of the decision function, and *ξ_i_* is a slack variable to compensate for any non-linearly separable training points.

The output is a reduced set of those training data, because most training data **x_i_** would have *α_i_* equal to 0. Those training examples which have non-zero *α_i_* are used as the final decision variables, and are called the support vectors (SV) [16].

Usually, four kernel functions are used in different cases. The RBF kernel non-linearly maps samples into a higher dimensional space, so it can handle the case when the relation between class labels and attributes is nonlinear. Compared to the RBF kernel, the polynomial kernel has more hyper-parameters, which influences the complexity of model selection. In addition, the sigmoid kernel behaves like RBF for certain parameters. Furthermore, there are some situations where the RBF kernel is not suitable. Thus, one may just use the linear kernel which is the simplest one. In this paper, the SVM detector uses two types of kernel functions to compare the performance with each other. The first is the simplest linear kernel, shown as:

(10) $$K\left(x_{i},x_{j}\right)=x_{i}^{T}x_{j}$$

and the second is a more popular radial basis function (RBF) kernel, shown as:

(11) $$K(x_{i},x_{j})=\exp\left(-\gamma {\left\|x_{i}-x_{j}\right\|}^{2}\right),\gamma >0$$

where *γ* controls the width of the function.

#### 3.2.2. Feature Extraction

**Figure 5.**
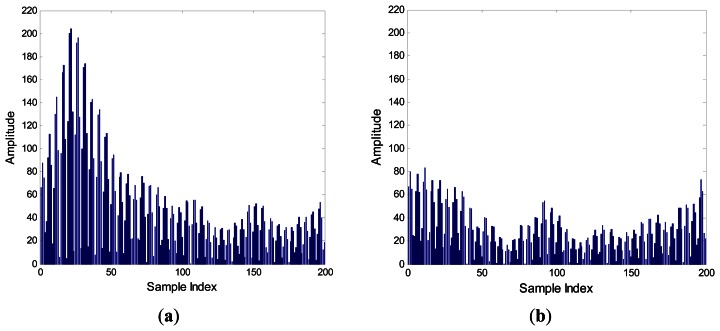
The SIF output of symbol “1” and “0”, respectively, in (**a**) and (**b**) for SNR = 0 dB.

The optimal selection of discriminant features is an issue of the greatest importance in EBPSK system. Figures 5(a,b) show the SIF output waveforms of “0” and “1”, respectively. The certain distinguishing features between the waveforms of “0” and “1” are very apparent. In order to establish the characteristics space that can identify two symbols, we may use the features as follows:

(1) If we define the area below y(n) as the energy, then we may note this energy item is quite concentrated at the left range of the characteristic waveform “1” when the received signals pass through the SIF. This energy is relatively dispersed while the symbol “0” passed through the SIF with only channel noise. As a result, we define the first feature as:
  (12) $$f_{1}=\frac{\sum_{n=s-p}^{s+p}y^{'2}(n)}{\sum_{n=1}^{M}y^{'2}(n)}$$
  where *M* = *N* × *f_s_*, *f_s_* is the sampling rate and the range of interest in (12) is limited by the key parameter P. Practically, P can be determined by the impacted part of received signals y′(n), which have been processed by taking the envelopes, as is shown in Figure 3. That is, P can be immediately obtained once the right value y′(n + k) has surpassed the left value y′(n) by δ. A simple and practical strategy is directly set δ to 0. On the other hand, if we use short bit duration then we can choose P = M/4, which means the half of most concentrated part of y′(n) are used. To make sure there are enough sampling points, we use P = 2N while P < 2N.
(2) It is noticeable that in Figure 5(a), the change rate of the characteristic waveform in the left range is much faster than that in Figure 5(b). Consequently, the variance in this range is also supposed to be much distinctive, so the second feature can be define as:
  (13) $$f_{2}={\sum_{n=S-P}^{S+P}\left(y(n)-\frac{1}{2S+1}\sum_{S-P}^{S+P}y(n)\right)}^{2}$$
(3) From Figure 5(a), the energy also exhibits a remarkable imbalance during the outside range. Specifically, the left range energy is much larger than the right, while these two parts are basically equivalent in Figure 5(b). Therefore, we can reasonably adopt this imbalance property as the third feature:
  (14) $$f_{3}=\sum_{n=S-P}^{S+P}y\prime (n)-\sum_{n=M-2P}^{M}y\prime (n)$$
  But, if we use short bit duration, the imbalance property is not as remarkable as the long bit duration ones. In this case, we can define the third feature as (15), which only uses the waveform property:
  (15) $$f_{3}^{\prime }=\sum_{n=1}^{M}y\prime (n)$$
(4) The total received energy can be also utilized to differentiate the two symbols, Therefore, we add it to our feature set as the fourth feature:
  (16) $$f_{4}=\sum_{n=S-P}^{S+P}y^{2}(n)$$

By taking full advantage of the developed characteristic waveforms, we have constituted a feature set which is dedicated to separating the two symbols. It is noteworthy that we do not need to estimate the channel noise power *σ*^2^, and choose only four features of SVM for training and testing, which can reduce the complexity significantly.

#### 3.2.3. The Detection Procedure of SVM

Based on above elaborations, we have taken full advantage of the EBPSK waveform and established a quantitative feature set Fl = [*f_1_*, *f_2_*, *f_3_*, *f_4_*] for long bit duration and Fs = [*f_1_*, *f_2_*, *f_3_*′, *f_4_*] for short bit duration, respectively, which is dedicated to separate the symbols “0” and “1”. Then, the detection of received signals can be formulated as determining a separating hyper-plane which divides the two-group pattern objects in a multidimensional features space, under the minimum classification errors. The SVM technique for EBPSK signal detection can be depicted as below:

##### Step 1: Features selection

An appropriate selection of discriminant features is carried out in order to determine the best performing features for the signal detection as Fl and Fs. By using the method, the original higher-dimensional inputs (the number of sampling points is M) will be transformed into lower-dimensional features. In our scheme, four remarkable features are chosen to separate the symbols “0” and “1”.

##### Step 2: Scaling

Scaling before applying SVM is very important. The main advantage of scaling is to avoid attributes in greater numeric ranges dominating those in smaller numeric ranges. Another advantage is to avoid numerical difficulties during the calculation. Because kernel values usually depend on the inner products of feature vectors, e.g., the linear kernel and the polynomial kernel, large attribute values might cause numerical problems. We use linearly scaling each attribute to the range [0,1].

##### Step 3: The training phase

The initial training stage only needs to be performed once unless the channel condition has varied significantly. Some training examples are given to the machine to create certain decision functions in order to differentiate the different types of objects, or so-called classes.

##### Step 4: The testing phase

During the testing stage, the SVM detector is ready for estimating the source bit based on classifying an unforeseen object, which is a new noisy data stream, and then classified by those decision rules. The detection task then becomes a pattern classification problem. The transmitted message bit is estimated by making a hard-decision from the decision function formed earlier in (7). The complexity of the SVM on the testing stage is independent on the number of symbols, but rather on the number of features per SV.

### 3.3. Channel Coding

#### 3.3.1. SVM Posterior Probabilities Estimate

We have made a hard-decision by using SVM classification, in some cases, such as channel decoder needs a posterior probability to achieve capacity. Platt has proposed that the SVM output can be transformed into posterior probabilities [24]. The method squashes the SVM soft output through a trained sigmoid function to predict posterior probabilities as follows:

(17) $$p(y=1|\text{x})\approx P_{A,B}(f)=\frac{1}{1+\exp(Af+B)}$$

where *f* = *f*(**x**), let each *f_i_* be an estimate of *f*(**x***_i_*). The best parameter setting z* = (A*, B*) is determined by solving the following regularized maximum likelihood problem:

(18) $$\underset{z=(A,B)}{\min}F(z)=-\sum_{i=1}^{l}\left(t_{i}\log(p_{i})+(1-t_{i})\log(1-p_{i})\right)$$

where *p_i_* = *P_A,B_*(*f_i_*), *t_i_* = (*y_i_* + 1)/2.

Unfortunately, log and exp could easily cause an overflow. If *p_i_* is near zero or *Af_i_* + *B* is large, exp(*Af_i_*+*B*) → ∞ and $1\;-p_{i}=1-\frac{1}{1+\exp(Af_{i}+B)}$ is a “catastrophic cancellation” when *p_i_* is close to one. The problem can usually be resolved by reformulation [25] and we can get the SVM PPEs as follows:

(19) $$p(y=1|\text{x})\approx \{\begin{array}{cc} \frac{1}{1+\exp(Af+B)}, & Af+B<0\\ \frac{\exp(-Af-B)}{1+\exp(-Af-B)}, & Af+B\geq 0 \end{array}$$

From (19) we can see that the PPE is an approximate one. This means that the SVM does not provide PPEs and its output needs to be transformed, before it can be interpreted as posterior probabilities.

#### 3.3.2. LDPC Coding

We employ low-density parity-check (LDPC) codes [26] to add redundancy to the transmitted binary sequence. LDPC codes have recently attracted a great deal of research interest, because of their excellent error-correcting performance and linear complexity decoding. Binary LDPC codes are now known to be capacity approaching on various channels when the block length tends to infinity. LDPC codes can be decoded by an iterative message-passing (MP) algorithm which passes messages between the variable nodes and check nodes iteratively. If the messages passed along the edges are probabilities, then the algorithm is also called belief propagation (BP) decoding, which is the optimal if there are no cycles or cycles are ignored. We propose to measure the BER performance of EBPSK system by using SVM detector with posterior probability output after a LDPC channel decoder has detected the received sequence. The procedure of LDPC decoding is as follows:

1. Initialization:
  (20) $$p_{n}^{0}(x)=q_{nm}^{0}=p(x_{n}=x|y_{n})$$
  where *p*(x_n_ = x | y_n_)is the PPEs of detector outputs.
2. Horizontal Step: the MAP output from *c_m_* to *ν_n_*:
  (21) $$\begin{array}{ll} r_{mn}^{k}(0)=p\left(\nu_{n}=0|c_{m}=0,y_{i\in \;B(m)\backslash n}\right),\\ r_{mn}^{k}(0)=\frac{1}{2}+\frac{1}{2}\prod_{i\in B(m)\backslash n}\left(1-2q_{im}^{k}(1)\right), & r_{mn}^{k}(1)=1-r_{mn}^{k}(0) \end{array}$$
3. Vertical Step: updating the message from *ν_n_* to *c_m_*:
  (22) $$\begin{array}{cc} q_{nm}^{k+1}(0)=\theta p_{n}^{0}(0)\;\prod_{j\in A(n)\backslash m}r_{jn}(0), & q_{nm}^{k+1}(1)=\theta p_{n}^{0}(1)\;\prod_{j\in A(n)\backslash m}r_{jn}\;(1) \end{array}$$
  *θ* is chosen to ensure $q_{nm}^{k+1}(0)+q_{nm}^{k+1}(1)=1$, Compute $p_{n}^{k}(x)$:
  (23) $$\begin{array}{cc} p_{n}^{k+1}(0)=\theta p_{n}^{0}(0)\;\prod_{j\in A(n)}r_{jn}(0), & p_{n}^{k+1}(1)=\theta p_{n}^{0}(1)\;\prod_{j\in A(n)}r_{jn}(1) \end{array}$$
4. Tentative output:
  (24) $$\nu_{n}^{k+1}=\{\begin{array}{cc} 1, & p_{n}^{k+1}(1)\geq 0.5\\ 0, & p_{n}^{k+1}(1)<0.5 \end{array}$$
  if all parity check equations are satisfied or max iterative number is reached, then stop iteration, else return to step 2.

Although, for classic digital modulation technologies the BP decoding is analyzed in [27], for the EBPSK system the SIF causes more difficulty in obtaining the posterior probability for LDPC decoding. In this paper, we focus on the initialization step for the posterior probabilities obtained by a nonlinear detector.

## 4. Simulation

In this part, we evaluate the performance of the proposed SVM detector and its soft output for LDPC decoding. For all simulations, unless specified otherwise, the system had 3,000 random symbols for training and the reported BER are computed using 10^6^ symbols and we average the results over 1,000 independent trials with random training and test data. We use *K* = 2, *A* = *B* = *1*, *θ* = *π* as the parameters of EBPSK modulation. The PPEs obtained by the SVM method are used as soft-input of the LDPC decoder. We measure the BER performance of the EBPSK system after the sequence has been corrected by LDPC decoder. During simulations, we use a 1/2 rate regular LDPC code with 1,000 bits per codeword and three ones per column.

### 4.1. Kernel Selection and Complexity Reduction

**Figure 6.**
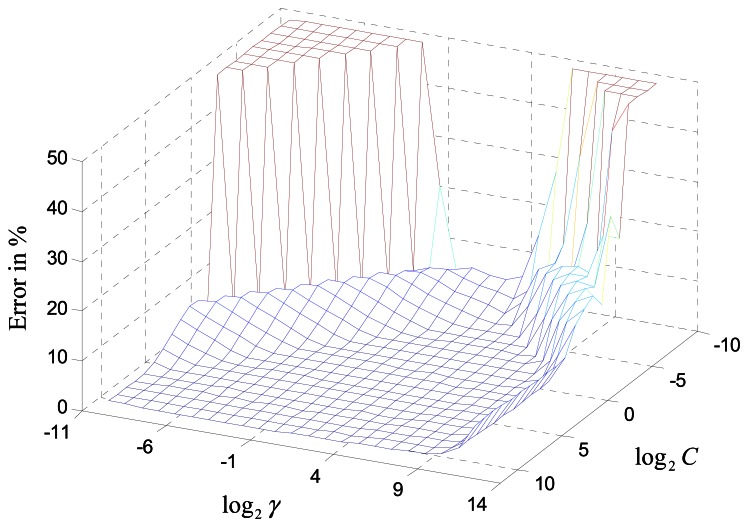
Cross-validation result of the SVM detector in RBF kernel.

In this subsection, the performance of the SVM detector, using the kernel functions (10) and (11), introduced in Section 3, is compared. The 10-fold cross-validation sweep from the training samples was used to find the optimum parameters of C and *γ* for the RBF kernel. Figure 6 shows that the width *γ* has a more dominating effect on the error rate than the penalty parameter C.

When *γ* is between 0.5 and 16, the SVM receiver has the best performance, regardless of the C parameter. A similar search was conducted for the linear kernel, but which only has the C parameter to adjust. Table 1 summaries the optimum SVM model obtained after the parameter search. Compared to the RBF kernel, the linear kernel has less SVs, which means the latter has a less computational complexity and thus would perform faster. In order to compare the BER performance fairly, both kernels used by the SVM detector were classifying exactly the same received signals.

**Figure 7.**
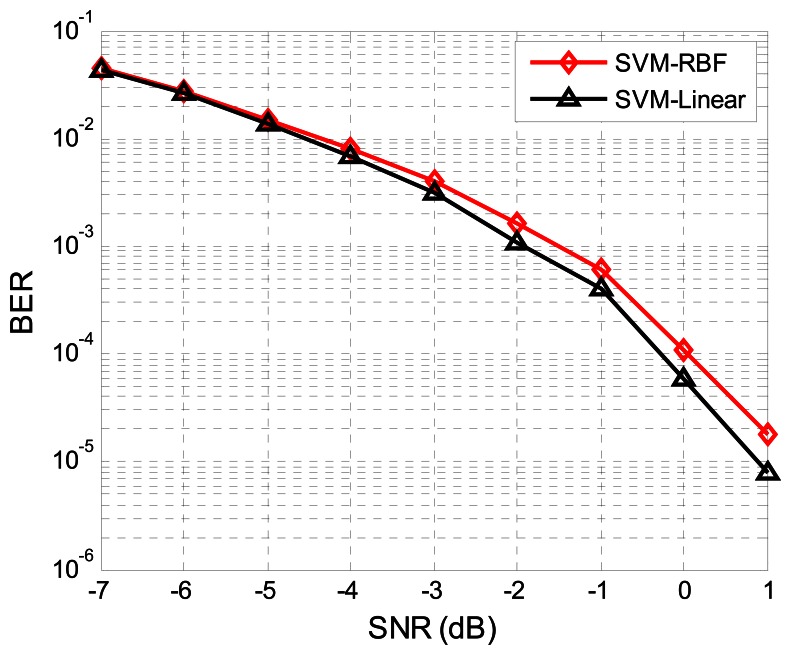
The BER performance of the SVM detector in different kernels. We use ◊ for the SVM with RBF kernel and Δ with linear kernel.

Figure 7 shows the BER performance of the SVM detector when employing different kernels. Although, the linear kernel is much simpler, its performance is slightly better than the RBF kernel. Therefore, we use a linear SVM kernel for the task. We train on a low SNR scenario (SNR = −7 dB in this case), proving that the SVM receiver does not need frequent re-training in different SNRs.

We have analyzed the BER performance of the SVM detector with linear kernel which is superior to the RBF one. Nevertheless, the solution for such a problem is computationally complex by consuming a mass of energy. The complexity of training an SVM for binary classification is O(n2), using the sequential minimal optimization [28]. However, the SVM detector should be analyzed for the testing stage only because the training time is very small compared with the actual testing time. A great amount of complexity can be reduced further if in the decision function given in (7) we use the linear kernel. The expression is simplified as follows:

(25) $$\begin{array}{ll} f(x) & =\sum_{i=1}^{L}\alpha_{i}y_{i}K(\text{x},{\text{x}}_{i})+b=\sum_{i=1}^{L}\alpha_{i}y_{i}(x_{i}^{T}x)+b\\ & =\left[\sum_{j=1}^{z}y_{j}\alpha_{j}\left(\sum_{i=1}^{n}x_{j,i}x_{i}\right)\right]+b=\sum_{i=1}^{n}x_{i}\left(\sum_{j=1}^{Z}y_{i}\alpha_{i}x_{j,i}\right)+b\\ & =\sum_{i=1}^{n}A_{i}x_{i}+b \end{array}$$

where Z is the number of support vectors, the constants $A_{i}=\sum_{j=1}^{Z}y_{i}\alpha_{i}x_{j,i}$ and b can be pre-computed before the testing stage to save computation time and energy. Therefore, the complexity of the SVM detector is *O*(*n*).

### 4.2. Detection

**Figure 8.**
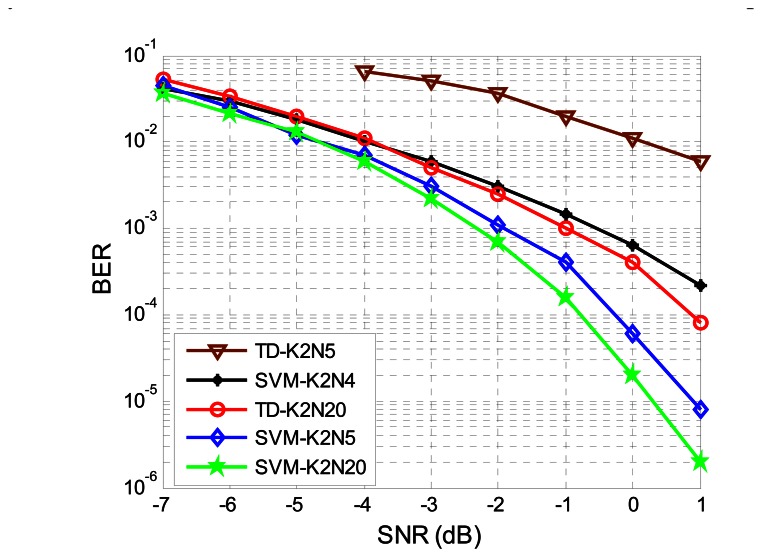
The BER performance of SVM detector with difference bit duration. We represent the threshold decision for bit duration N = 5 with ▼) and N = 20 with ○, respectively; the SVM for N = 4 with *, N = 5 with ◊ and N = 20 with ✩ , respectively.

The BER performance of the EBPSK system will be diverse with different bit durations and sampling rates. To prove the effectiveness of the proposed method, various simulations were conducted. In Figure 8 we compare the BER performance of the SVM detector with TD for different bit durations N. The SVM-K2N5, SVM-K2N4, SVM-K2N20 and TD-K2N20 BER plots in Figure 8 perform significantly better than TD-K2N5. Compared to SVM-K2N5, the performance of SVM-K2N4 deteriorated greatly, and the former performed slightly worse than the SVM-K2N20. On the one hand, we should use N > 4 in order to get the desired performance; on the other hand, the shorter the bit duration N the higher the data rate. Thus, N = 5 is the best choice for our system with the SVM technique. Unless specified otherwise, all simulations assume N = 5. Moreover, we can appreciate that the BER performance of SVM-K2N5 is even better than that of TD-K2N20. This means that the performance of conventional TD is greatly affected in the case of short bit duration N. In this sense, SVM detector with higher bit rate outperforms the TD.

**Figure 9.**
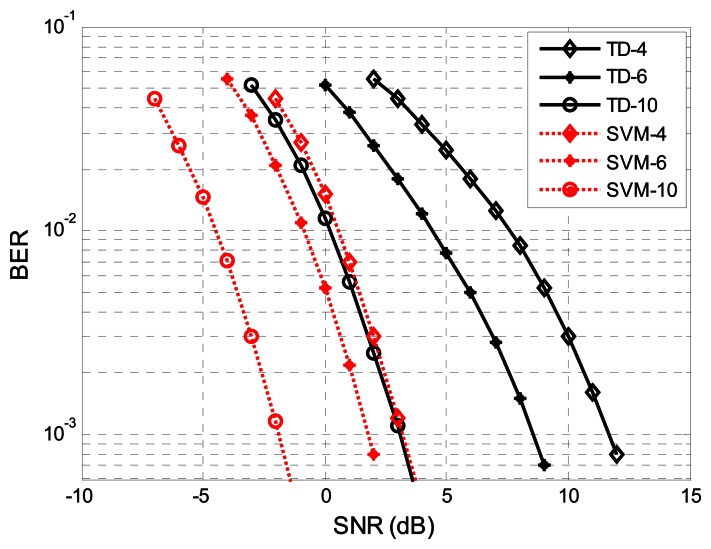
The BER performance comparison of the SVM with threshold decision by different sampling rate. We use dashed-dotted lines for the SVM BER, solid lines for the threshold decision BER. We represent the BER for *f_s_* = 4*f_c_* with ◊, *f_s_* = 6*f_c_* with *, and *f_s_* = 10*f_c_* with ○, respectively.

The BER performance comparison of the SVM with TD by different sampling rates is plotted in Figure 9. Compared to the TD, the SVM method can upgrade more than 8 dB, 7 dB and 5 dB for *f_s_* = 4*f_c_*, *f_s_* = 6*f_c_* and *f_s_* = 10*f_c_* at BER = 10^−3^, respectively. This means the performance of the SVM detector improved significantly while the sampling rate is low, and it is more robust to sampling rate than TD.

In the next experiment we face a bandwidth efficient communication model which is proposed via a NBPF at the transmitting end of the system. Though we can achieve a bandwidth efficient transmission and suppress the interference to other channels, the transmitted signals would be distorted and the amplitude of SIF output signal would be not as high as usual. We use the SVM detector to solving such issue and give the BER performance comparison between SVM detector and TD. The bandwidth of the linear phase NBPF is designed to be $\left[\frac{0.98N}{T},\frac{1.02N}{T}\right]$.

**Figure 10.**
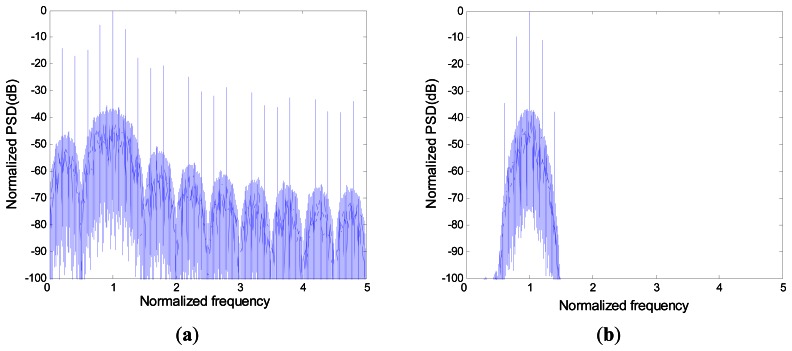
The power spectrum density of modulated signals and its filtered signals, respectively, in (**a**) and (**b**) for N = 5.

PSD of the modulated signals is plotted in Figure 10(a). When this signal is filtered by the NBPF, its corresponding spectrum is illustrated in Figure 10(b).

**Figure 11.**
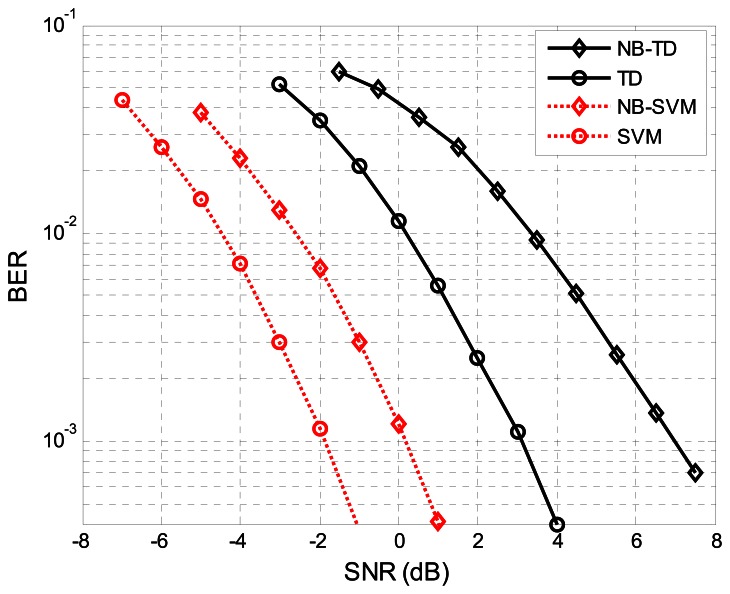
The BER performance comparison of SVM and threshold decision for general system and narrow band system with N = 5. We use dashed-dotted lines for the SVM BER, solid lines for the threshold decision BER. We represent the BER of narrow band system with ◊) and general system with ○, respectively.

The performance comparison of SVM detector and TD are presented in Figure 11. The SVM detector can improve the quality of the receiver significantly. For the SVM, the SNR gain over the NB-SVM is around 2 dB with the BER = 10^−3^ and for the TD, the gain over the NB-TD is around 4 dB with the BER = 10^−3^.

This can be explained by the fact that when the signal was filtered by the NBPF it was distorted significantly and it was difficult to detect the signal through TD, but for the SVM detector which can make full use of the characteristics of SIF output signals, such as energy and waveforms, thus the SVM-NB is about 2 dB from the performance achieved by the SVM. Moreover, the NB-SVM is even better than TD and the former outperforms the latter by about 3 dB and outperforms the NB-TD by 7 dB with the BER = 10^−3^. This demonstrates that the performance can be improved by using the SVM detector in a band efficient transmission system. We have shown that there are more advantages in the SVM method than TD and the BER performance was significantly improved by the former compared with the latter. Moreover, the SVM method is not as sensitive to the sampling rate as the threshold method. Thus, SVM is an effective method for EBPSK detection.

### 4.3. Channel Coding

In previous subsection, we have discussed the detector based on SIF together with SVM classifier, when we compare performances at low BER. In this section, we focus on the performance after the sequence has been corrected by an LDPC decoder and the ability of SVM detector to provide accurate posterior probability estimates instead of measuring the performance of the demodulator at low BER, because the channel decoder can achieve those BER values at significantly lower signal power. Earlier this year a study has been undertaken to give the approximate LLR for LDPC decoding [29], so we now modify the method to provide posterior probability estimates and the method is referred to as modified approximate posterior probability estimates (MAPPE) in this paper.

**Figure 12.**
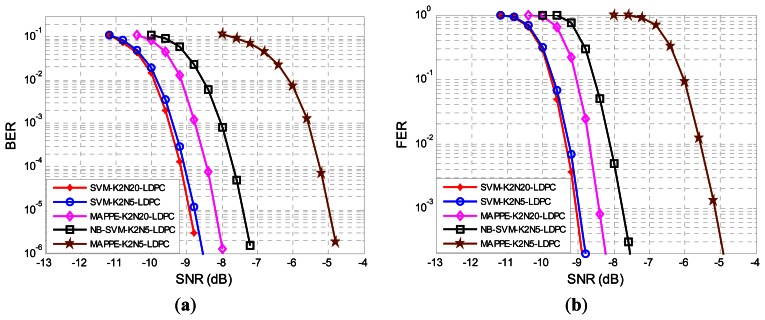
We plot the BER performance at the output of LDPC decoder with soft-inputs using a SVM and MAPPE detector with N = 5, N = 20 and narrowband system in (**a**) and its corresponding FER in (**b**). We represent the SVM method for N = 20 with *, N = 5 with ○) and narrow band system with □, respectively; the MAPPE method for N = 5 with ✩ and N = 20 with ◊, respectively.

In Figure 12(a) we have depicted the BER for the MAPPE and SVM receiver. The BER can be significantly reduced by the SVM-K2N5-LDPC method compared to the MAPPE-K2N5-LDPC method. Also, we can appreciate that the SVM-K2N5-LDPC is even better than MAPPE-K2N20-LDPC. Therefore, the SVM method significantly reduces the BER at lower SNR, because SVM posterior probability estimates are more accurate than MAPPE and the LDPC decoder can rely on these trustworthy predictions. Although the SVM-K2N5-LDPC has a higher data rate than SVM-K2N20-LDPC, their performance are almost identical; this is a benefit of the SVM features extraction method which makes full use of the signal characteristics and narrows the difference between the long bit duration system and the short one. In what follows we only report results for the SVM-K2N5-LDPC for clarity purposes. In addition, we plot the BER performance of narrowband system that applied LDPC codes by using the soft-input of SVM detector output. The BER performance of NB-SVM-K2N5 outperforms the MAPPE-K2N5-LDPC by 2.5 dB and is less than 1 dB from the MAPPE-K2N20. For completeness, we have also depicted the FER in Figure 12(b). The FER performance is typically used when we are only interested in error-free frames, which is a more relevant measure in data-package networks. The results in FER are similar to the BER ones.

**Figure 13.**
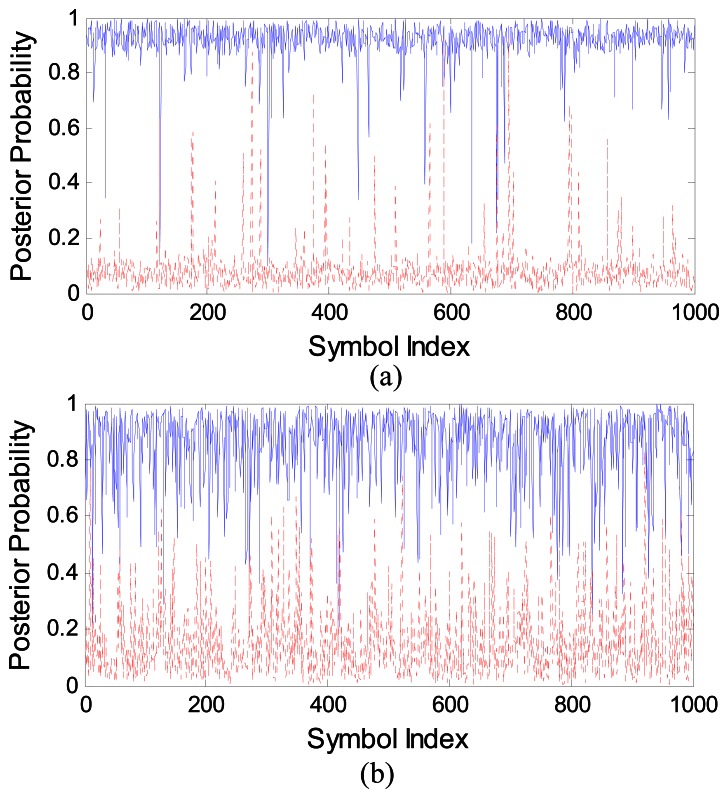
We plot the SVM PPE and MAPPE, respectively, in (**a**) and in (**b**).

To understand the difference in PPEs, we have plotted the curves for the SVM and the MAPPE in Figures 13(a,b), respectively, with bit duration N = 5 and SNR = −5 dB. We depict the estimated probabilities P(y = 1 | x) with the true ones and P(y = 0 | x) with 1-P(y = 0 | x) for clarity purposes. Figure 13(a) shows that the SVM PPEs are closer to “1” (or “0”) and less spread, most of the values of SVM detector output are between 0.9 and 1 for symbol “1” (or between 0 and 0.1 for symbol “0”). Thereby, SVM estimates are closer to the true posterior probability, which explains its improved performance with respect to the MAPPE, when we measure the BER after the LDPC decoder.

**Figure 14.**
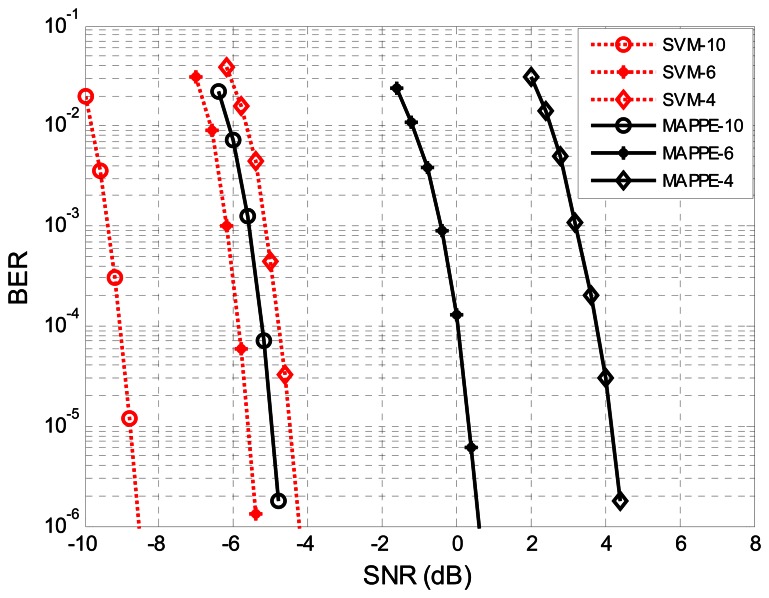
The BER performance at the channel decoder with SVM PEEs (dashed line) and with MAPPE (solid line). We represent the BER for *f_s_* = 4*f_c_* with ◊, *f_s_* = 6*f_c_* with *, and *f_s_* = 10*f_c_* with ○, respectively.

In the last experiment we will compare the ability to provide PPEs between SVM and MAPPE with different sampling rates. In Figure 14 we have depicted the BER for the two receivers. The BER performance of the two receivers follows the same lines that we have seen in the previous detection case: the SVM performs well and outperforms the MAPPE. Compared to the MAPPE-LDPC, the SVM-LDPC can upgrade more than 8.5 dB, 6 dB and 4 dB for *f_s_* = 4*f_c_*, *f_s_* = 6*f_c_* and *f_s_* = 10*f_c_*, respectively. This means that the performance of SVM-LDPC is improved significantly when the sampling rate is low, and it is not sensitive to the sampling rate. Also, Figure 14 illustrates that the SVM detector is superior than the MAPPE in bad conditions; this means that the more accurate PPEs can be obtained by the SVM detector at low sampling rates than with the MAPPE method.

## 5. Discussion and Conclusions

In this paper, we introduced a new approach for nonlinear detection based on a SVM classifier. A simulator of the system with high data rate and high spectra efficiency was designed. We show that the performance can be significantly improved by using a linear SVM kernel for detection, which has less computational complexity and thus saves computation time and energy. Moreover, we only use four features for training and testing, which makes full use of the characteristics of SIF output signals and reduces the complexity significantly. Furthermore, we concentrated not only on reducing the BER of detection, but also on providing accurate PPEs. The BER performance was significantly improved for the SVM-LDPC compared with the MAPPE-LDPC approach. Also, the SVM method is more robust to sampling rate than the MAPPE method, and the former is proposed for use in detection when the sampling rate is low. In addition, we analyzed the SVM detector for a bandwidth efficient communication system with the NBPF added at the transmitting end. Such a system can meet the requirements of bandwidth limitation and achieve the desired performance. As a by-product, we have shown that the short bit duration (*i.e.*, N = 5) makes the ISI, but the performance is almost identical when measured after the LDPC decoder. Also, the SVM probability output method does not need to estimate the channel noise power *σ*^2^, which reduces the complexity.

In fact, the features selection procedure is somewhat elementary as an early work, and we haven't considered the resort to the feature combination technique which can reduce the corresponding problem dimensiond. If the optimal feature combination is used, accompanying the well-established features selection procedure, the gains achieved with this technique can be further enhanced, which also remains as an attractive area for future research.

## Figures and Tables

**Table 1.** Comparison of SVM models.

	**Selected Kernel**	
	RBF	Linear
C	8	2
*γ*	8	-
SVs	462	381
